# Perspective: Observational Studies Involving Low-Soy Intake Populations Have Limited Ability for Providing Insight into the Health Effects of Soybean Isoflavones

**DOI:** 10.1016/j.advnut.2024.100210

**Published:** 2024-03-12

**Authors:** Mark J Messina, Virginia Messina, Chisato Nagata

**Affiliations:** 1 Soy Nutrition Institute Global, Jefferson City, MO, United States; 2 Nutrition Matters, Inc., Pittsfield, MA, United States; 3 Department of Epidemiology and Preventive Medicine, Gifu University Graduate School of Medicine, Gifu, Japan

**Keywords:** soy, isoflavones, epidemiology, clinical trials, causality, associations, biological plausibility, intake recommendations

## Abstract

Isoflavones are naturally occurring plant compounds found in uniquely high amounts in soybeans and foods made from this legume. These soybean constituents have been proposed to exert several health benefits and as such they have been the subject of an enormous amount of research. This research includes randomized controlled trials (RCTs) and epidemiologic investigations. Although statistically significant associations between isoflavone intake and a wide range of health outcomes have been identified in cohorts involving low-isoflavone intake populations, we suggest that these associations are unlikely to have a causal basis because exposure is too low for isoflavones to exert physiologic effects. In cohorts involving predominantly non-Asian, non-vegetarian populations, the highest isoflavone intake category is typically ≤3 mg/d, an amount of isoflavones provided by ∼30 mL (2 tablespoons) of soymilk made from whole soybeans. In comparison, mean isoflavone intake in the upper intake categories in observational studies involving high-isoflavone intake populations is typically ≥50 mg/d. In RCTs, intervention doses of isoflavones typically range between 40 and 100 mg/d. Health professionals advising patients and clients about soy food and isoflavone intake need to be aware of the limitations of epidemiologic research involving low-isoflavone intake populations. Intake recommendations are best based on the results of RCTs using clinically relevant doses of isoflavones and epidemiologic studies involving populations for whom soy foods are a habitual part of the diet.

**Statement of Significance**

Statistically significant associations between soybean isoflavones and a wide range of health outcomes have been identified in cohorts involving low-isoflavone intake populations. We suggest that these associations are unlikely to have a causal basis and therefore, do not provide insight into the health effects of soy foods or isoflavones. Soy and isoflavone intake recommendations are best based on randomized controlled trials and epidemiologic studies involving populations whose habitual diet includes soy foods.

Observational studies have greatly informed understanding the of diet and health relationships [[1], [2], [3]]. However, epidemiologic insight into the health effects of soybean isoflavones is constrained by the limited number of cohorts that provide a clinically relevant isoflavone intake range. Those that do provide relevant intakes primarily involve participants from Asian countries or large numbers of vegetarians/vegans. Isoflavones are naturally occurring plant compounds present in a wide range of commonly consumed foods but in only negligible amounts compared with soybeans [4,5]. Genistein, daidzein, and glycitein and their respective glycosides, account for ∼50%, 40%, and 10% of the total isoflavone content of the soybean, respectively [6]. These diphenolic molecules are the subject of a large amount of research as >1000 isoflavone-related papers are indexed in PubMed annually.

Research on the effects of isoflavones on health includes randomized controlled trials (RCTs) using soy foods, soy protein, or isoflavone supplements and observational studies of usual isoflavone intake from food. Observational studies in Asian populations involve a wide range of isoflavone consumption based on varying intakes of soy foods. In contrast, observational studies involving low-soy intake populations, which include most non-Asian populations, involve much lower isoflavone intakes and thus, a much smaller intake range [[7], [8], [9], [10], [11]]. In this paper, we suggest that the utility of these studies for providing insight into the health effects of isoflavones (and indirectly soy foods) is questionable because isoflavone intake is too low and intake ranges too small to assess reasonable and relevant dose–response relationships. This concern applies to studies of low-isoflavone intake populations that find statistically significant effects of isoflavone intake as well as those that do not. Dietitians and other health professionals who counsel clients and patients about soy intake should be aware of the limitations of this epidemiologic research regardless of whether statistically significant associations are found. Although the focus of this paper is on isoflavones, because soybeans are uniquely rich sources of these compounds, the points made in this perspective also have bearing on understanding of the health effects of soy foods.

## Isoflavones and Health: Brief Overview

Isoflavones are commonly classified as phytoestrogens [[12], [13], [14], [15]] and, although to a lesser extent, as selective estrogen receptor modulators [14,[16], [17], [18]]. However, the degree to which isoflavones exert hormonal effects in men or women is unclear [19,20]. The classification of isoflavones as phytoestrogens is based primarily on in vitro [21,22] and animal [[23], [24], [25], [26]] data. Isoflavones have been investigated for several health benefits including reducing the risk of breast cancer [27,28], improving cognitive function [29] and arterial health [30], alleviating menopausal symptoms [31], and reducing wrinkle severity [32]. Conversely, several safety concerns have been raised about these soybean constituents [[33], [34], [35]].

There are ∼3–4 mg isoflavones per gram protein in traditional Asian soy foods, such as tofu, tempeh, and miso [36,37]. (Throughout this paper, isoflavone amounts refer to the aglycone equivalent weight.) Thus, as a rough guide, one serving, such as 240 mL soymilk made from whole soybeans, contains ∼25 mg isoflavones [38]. However, as a result of processing losses, concentrated sources of soy protein, such as soy protein isolate and soy protein concentrate, which are ∼90% and 65% protein, respectively [39], typically have a more variable and lower (≤2 mg/g protein) isoflavone content than traditional Asian soy foods [4,6,40].

## Isoflavone Intake among Populations

There are no official isoflavone intake recommendations from governmental agencies or well-established health organizations although the Chinese dietary guidelines specifically call for consuming soybeans and soy products [41]. In Japan, older adults typically consume 30–50 mg/d isoflavones [36,37,42,43], whereas in China, soy intake varies considerably among geographic regions [44,45]. For example, mean isoflavone intake in rural Chinese women was found to be only ∼18 mg/d [44], whereas among Shanghainese men [46] and women [47], it is closer to 40 mg/d. A small percentage of individuals in Japan or China consume >100 mg/d [[46], [47], [48]]. In comparison, United States [49], Canadian [50] and European [51] per capita isoflavone intake is typically <3 mg/d.

## RCTs Involving Isoflavone Interventions

RCTs hold the most potential to elucidate how isoflavone exposure affects health. In RCTs involving isoflavone interventions, participants generally consume between 40 and 100 mg/d provided as supplements, soy protein or soy foods, although there are notable exceptions wherein much higher doses were consumed. For example, of the 19 RCTs (20 intervention arms) included in a meta-analysis evaluating the efficacy of isoflavones for alleviating menopausal hot flashes, the intervention dose was within the 40–60 mg/d range in 13 intervention arms, whereas it was higher in 2 intervention arms and modestly lower in 5 intervention arms [31]. It was concluded that the efficacious isoflavone dose is ∼50 mg/d. Similarly, in a meta-analysis of 9 RCTs that evaluated the effect of isoflavones on endothelial function, all intervention doses were between 40 and 100 mg/d [30]. Isoflavones were found to improve endothelial function in postmenopausal women with impaired endothelial function at baseline. Finally, a meta-analysis of 16 RCTs that evaluated the impact of isoflavones on cognitive function included 12 interventions with doses between 60 and 100 mg/d and 4 interventions using >100 mg/d [29]. The authors concluded that isoflavones may improve cognitive function in older adults.

Concerning selected examples of very high-isoflavone doses, RCTs that evaluated effects on bone mineral density in postmenopausal women intervened with 300 mg/d in 1 study [52] and 200 mg/d in another [53]. One 5-y RCT that examined effects on endometrial tissue intervened with 150 mg/d isoflavones [54] and a 6-mo RCT evaluating the effects on breast cell proliferation intervened with 235 mg/d, 150 mg of which was genistein. Finally, although interventions typically range in duration from 4 wk to 6 mo, several longer term (≥2 y in duration) RCTs have been conducted [[54], [55], [56], [57], [58]].

## Observational Associations Involving Low-Isoflavone Intake Cohorts Are Likely Spurious

Over the past 25 y, many observational studies involving populations with clinically relevant isoflavone intakes have examined a wide range of health outcomes (see references for selected examples) [43,47,[59], [60], [61], [62], [63], [64]]. However, many epidemiologic studies involving low-isoflavone intake populations (see references for selected examples) have also examined the health effects of isoflavones [11,[65], [66], [67], [68], [69], [70], [71], [72], [73], [74], [75], [76], [77], [78], [79], [80], [81]]. Some of these studies find benefits, such as reductions in risk of all-cause mortality [65], metabolic syndrome [78], prediabetes [79], depressive symptoms [80], osteoporosis [81], and metabolic associated fatty liver disease [67].

Nevertheless, we maintain that the results of these investigations are of limited utility for helping to understand the health effects of isoflavones, and by extension, soy foods. As far back as 2004, skepticism about the utility of this research was raised [82]. In our opinion, it is biologically implausible that such negligible amounts of, or differences in isoflavone consumption, cause differences in health outcomes, and thus, the associations may be occurring by chance or due to other confounding factors. If this view is correct, these studies do not provide reliable insight into the health effects of isoflavones.

As shown in Table 1, in the upper intake category of the 10 studies involving high-isoflavone intake populations, isoflavone intake generally ranges between 50 and 60 mg/d [37,43,48,[83], [84], [85], [86], [87], [88]]. These amounts match the intervention doses typically used in RCTs. In contrast, in the upper intake category of the 9 studies involving low-isoflavone intake populations, isoflavone intake is generally ∼1 mg/d [65,66,68,72,73,76,[89], [90], [91]]. There are ∼3 mg isoflavones in 30 mL (2 tablespoons) of soymilk made from whole soybeans. In one study conducted in the United States, listed in Table 1, the mean 5th quintile isoflavone intake was 2.5 mg/d for men and 1.5 mg/d for women [66]. Although these values are higher than the values for some low-intake populations, they are significantly lower compared with the intake levels in high-intake populations and the doses used in RCTs.

**TABLE 1:** Isoflavone intake in the upper intake categories in selected observational studies involving low- or high-isoflavone intake populations that examined the relationship between isoflavone intake and health outcomes

First author/reference	Country	Primary healthoutcome of interest	Highest intake category	Isoflavone intake (mg/d) in the highest intake category for men		Isoflavone intake (mg/d) in the highest intake category for women	
High-isoflavone intake populations							
Konishi et al. /[37]	Japan	Type 2 diabetes	3rd tertile	68.9^1^	35.4^2^	62.4^1^	32.4^2^
Shirabe et al. /[48]	Japan	Breast cancer	4th quartile	—	—	44.8^1^	23.1^2^
Nakamoto et al. /[83]	Japan	All-cause mortality	3rd tertile	46.1^1^		17.4^2^	
Chei et al. /[43]	Japan	Lung cancer	4th quartile	84^1^ (men and women)		37^2^ (men and women)	
Hara et al. /[84]	Japan	Gastric cancer	4th quartile	48.8^1^^,^^3^	0.2^4^	48.1^1^^,^^3^	0.03^4^
Wu et al. /[85]	China	Inflammation	5th quintile	—	—	59.6^1^	0.7^2^
Chei et al. /[43]	China	Lung cancer	4th quartile	60^1^ (men and women)		19^2^ (men and women)	
Yang et al. /[86]	China	Colorectal cancer	4th quartile			48.9^1^	NR
Yu et al. /[87]	China	Coronary heart disease	4th quartile	58.3^5^	NR	—	—
Jacobsen et al. /[88]	United States (SDA)	Nulliparity	∼90th percentile	—	—	79.3^1^	32.5^2^
Low-isoflavone intake populations							
Hedelin et al. /[72]	Sweden	Ovarian cancer	3rd tertile	—	—	0.233^1^	0.98–4.61^7^
Chen et al. /[65]	United States	All-cause mortality	5th quintile	2.5^1^	NR	1.5^1^	NR
Zhou et al. /[66]	United States	Mortality Alive Deceased	Overall	2.15^1^ (men and women) 1.08^1^ (men and women)		0.16^2^ (men/women) 0.30^2^ (men/women)	
Cotterchio et al. /[76]	Canada	Breast cancer	Overall	—	—	0.230	0.093, 0.842^6^
Rossi et al. /[89]	Italy	Endometrial cancer	Overall	—	—	0.0424^1^	0.0582^7^
Kuhnle et al. /[73]	UK	Bone mineral density	Overall	0.8755^5^	0.6435, 1.1989^6^	0.6198^5^	0.4581, 0.8362^6^
Wang et al. /[68]	United States	Cancer	3rd quartile 4th quartile	0.440 – 0.810 (men and women) >0.810 (men and women)			
Wu et al. /[85]	United States	Chronic respiratory diseases	75th percentile 95th percentile	0.04^1^ (men and women) 5.72^1^ (men and women)			
Liu et al. /[91]	United States	Periodontitis	3rd tertile	3.08^1^ ± 14.26^2^ (men and women)			

Abbreviations: NR, not reported; SDA, seventh-day adventists.

1 Mean.

2 SD.

3 Genistein only (men, daidzein: 29.7 ± 0.1; women).

4 SE.

5 Median.

6 IQR.

7 75th percentile.

For these reasons, we think that it is unlikely any identified associations in observational studies involving low-isoflavone intake populations have a causal basis. It is theoretically possible that when compared with the efficacious doses identified in RCTs, a much lower daily isoflavone intake over many years may produce health effects, but in our view, these intakes are too low for this to be the case. We are unaware of any intake recommendations for foods or nutrients that differ so dramatically from the amounts shown to produce effects in RCTs.

Furthermore, accurately quantifying the isoflavone intake of low-isoflavone intake cohorts is difficult because intake is coming from soy foods that are very infrequently consumed or from a wide range of foods that naturally contain minute amounts of isoflavones and from foods, such as bread, to which small amounts of soy protein have been added [92]. Lee et al. [93] have argued that isoflavone intake is underestimated because of the presence of soy in foods, sometimes referred to as “hidden soy” [94], not typically thought of as containing soy. This possible underestimation further complicates the utility of observational studies involving low-isoflavone intake populations.

The concept that epidemiologic associations derived from low-isoflavone intake populations have limited utility for providing insight into health effects has been noted by other researchers. A case in point is an analysis of the Study of Women’s Health Across the Nation, a prospective study that includes 5 racial/ethnic groups [95]. However, the analysis between genistein intake and cognitive function was limited to Chinese and Japanese women because according to the authors, only these ethnic groups had sufficient genistein intake for evaluation. Also relevant are 2 recently published meta-analyses of observational studies that found isoflavone intake is associated with a reduced risk of developing breast cancer [96,97]. However, this benefit was observed only in Asian, not in non-Asian studies, which in both cases, the authors attributed to the low-isoflavone intake among participants of non-Asian studies. Finally, it is instructive to consider the approach used by Guha et al. [98] and Caan et al. [99] in their observational analyses of the relationship between isoflavone intake and breast cancer recurrence among United States women. These authors created a 95th percentile intake as a separate category because they assumed that “ … any beneficial effect of soy would be seen only at a level similar to that consumed in Asian populations” [98]. Wu et al. [91] also created a 95th percentile isoflavone intake category but in this case, the mean was only 5.72 mg/d.

## Low-Dose Hypothesis

Unless one argues for the application of homeopathic principles [100], there must be an isoflavone dose that is too low to exert a biological effect. We are aware of the low-dose hypothesis, which maintains chemicals can cause a more significant biological response at low doses than high doses [[101], [102], [103]]. However, the relatively high suggested upper limits on isoflavone intake issued by a variety of governmental and nongovernmental agencies are unsupportive of this hypothesis [[104], [105], [106]].

Furthermore, the low-dose hypothesis is not applied to other foods and food bioactives, such as the impact of glucosinolates in cruciferous vegetables on thyroid function [107] or for that matter, to antinutrients in general [108]. In the case of isoflavones, the low-dose hypothesis could theoretically be evaluated clinically. In fact, to a limited extent, trials potentially relevant to the low-dose hypothesis have been conducted. Soy protein from which the isoflavones have been almost completely removed has served as the control in numerous clinical trials [[109], [110], [111], [112], [113], [114], [115], [116], [117]]. Evidence from these trials does not suggest such low-isoflavone doses exert health effects.

## Evaluating Evidence for Isoflavone Intake Recommendations

As outlined above, our view is that findings from observational studies of low-isoflavone intake populations likely do not provide a useful guide for soy food or isoflavone intake recommendations. Instead, we recommend that intake recommendations rely on 2 lines of evidence. These are the results of RCTs using clinically relevant doses of isoflavones and epidemiologic studies involving populations whose habitual diets include varying amounts of isoflavone-rich soy foods. Although soy intake varies widely among Asian countries and regions [[46], [47], [48],[118], [119], [120], [121], [122]], intake among older adults in Japan, which ranges from ∼1 to 3 servings daily of traditional soy foods, providing ∼25 to 75 mg isoflavones, can serve as one reasonable intake guide [37].

Finally, if epidemiologic studies involving low-isoflavone intake populations are conducted, we recommend that the authors of these studies articulate why the observed range of intake would plausibly demonstrate differences in outcomes. Given the number of cohorts involving high-intake populations and the number of RCTs that involve isoflavone interventions, it is still readily possible to gain insight into the health effects of isoflavones without relying on data from low-isoflavone intake populations.

In conclusion, dietitians and other health professionals who counsel clients and patients about soy should be aware that, regardless of the statistical significance of associations identified in epidemiologic studies, it is important to consider the intake levels of isoflavones. Associations in studies that involve low-intake levels may not have a causal basis. Although authors often acknowledge that the lack of an observed association might be a result of low exposure, this cautionary note is typically missing when associations are found. Furthermore, these low-intake population studies are part of the scientific literature and included in meta-analyses.

In our view, advice about incorporating soy foods into the diet is best based on findings from RCTs using relevant doses of isoflavones and observational studies involving populations whose habitual diet includes soy foods. Finally, because similar to all foods, soybeans contain multiple bioactives, the lack of effect of isoflavones on a particular health outcome, does not necessarily imply that soy foods will also be without effect.

## Author contributions

The authors’ responsibilities were as follows – MM: wrote the first draft with contributions from CN; all authors: reviewed and commented on subsequent drafts of the manuscript; all authors: participated in the literature review conducted for, and participated in the writing of, the manuscript; MM: was responsible for the response to reviewers; and all authors: reviewed and approved the final manuscript and the response to reviewers.

### Conflict of interest

MM is associated with Soy Nutrition Institute Global as an employee. All other authors report no conflicts of interest.

## Funding

The authors reported no funding received for this study.
